# Supplementary material for: The joint effect of weight-adjusted waist index and physical activity on all-cause mortality in Chinese elderly patients with multimorbidity: A study based on the CLHLS from 2011 to 2018
Source: PLoS One. 2025 Jun 9;20(6):e0325886. doi: 10.1371/journal.pone.0325886 (PMC12148107; doi:10.1371/journal.pone.0325886)
Supplement: S1 Table — (PDF) [file pone.0325886.s001.pdf]

S1. Interaction analysis

Variables	z	P>z	[95% conf. interval]
WWI	1.54	0.125	[0.963,1.359]
exercise	0.49	0.627	[0.878, 1.241]
interaction	-0.15	0.884	[0.890, 1.106]
