# Supplementary material for: The joint effect of weight-adjusted waist index and physical activity on all-cause mortality in Chinese elderly patients with multimorbidity: A study based on the CLHLS from 2011 to 2018
Source: PLoS One. 2025 Jun 9;20(6):e0325886. doi: 10.1371/journal.pone.0325886 (PMC12148107; doi:10.1371/journal.pone.0325886)
Supplement: S2 Table — (PDF) [file pone.0325886.s002.pdf]

S2. Moderating Effect

Variables	p	Exp(B)	[95% conf. interval]
Remain inactive			
Remain active	0.000	0.582	[0.488, 0.693]
Inactive to active	0.011	0.828	[0.716, 0.958]
Active to inactive	0.007	1.253	[1.063, 1.478]
WWI	0.017	1.152	[1.026, 1.294]
interaction	0.844	0.990	[0.896, 1.094]
