# Supplementary material for: The joint effect of weight-adjusted waist index and physical activity on all-cause mortality in Chinese elderly patients with multimorbidity: A study based on the CLHLS from 2011 to 2018
Source: PLoS One. 2025 Jun 9;20(6):e0325886. doi: 10.1371/journal.pone.0325886 (PMC12148107; doi:10.1371/journal.pone.0325886)
Supplement: Attachment — Submitted filename: Editor report Plos One Journal.pdf [file pone.0325886.s003.pdf]

Editor Comments:

1. Please check the manuscript for grammatical errors and awkward phrasing.

2. Material and method section: The exclusion of participants without multimorbidity prior to death is justified, but it would be helpful to provide more information on how "multimorbidity" was specifically defined or identified. Was it based solely on self-reports or was any medical documentation used to confirm diagnoses?

3. It would be useful to clarify whether participants who developed multimorbidity during the study were included or excluded.

4. The categorization of physical activity based on responses to the questionnaire seems to lack some nuance. For example, how were the activities coded as "yes" or "no"? Was it based on frequency, intensity, or duration of physical activity? Clarifying these details would improve the understanding of what "physical activity" means in the context of this study.

5. Discussion section: While the summary is effective, the section could provide a more detailed explanation of why these findings are important in the context of multimorbidity management. For example, while the study identifies the protective role of physical activity and increased risk of mortality due to WWI, a deeper exploration of how these results inform clinical practice or public health strategies for multimorbidity patients would be helpful.

Reviewer 1:

Giving a more convincing explanation of why the weight-adjusted waist index (WWI) should be used rather than more traditional metrics like BMI would improve the text.

Methodology:

Any explanations about the thresholds used should be included in the criteria for classifying WWI levels into "lower" and "higher" categories. This would improve reader comprehension and reproducibility.

It would be beneficial to have more information on the confounders that are adjusted for in the Cox proportional hazards models. Were lifestyle and socioeconomic factors fully controlled for?

The findings imply that regular exercise lessens the detrimental effects of high WWI mortality. It could be worthwhile to talk about potential explanations for this protective effect, such how exercise can lower inflammation or enhance metabolic health.

Limitations and Discussion:

Even if the study emphasizes the importance of physical exercise, it would be helpful to examine why changing from an active to an inactive state raises the risk of death. Could this be because people stopped exercising because their health deteriorated?

Recognize that using self-reported physical activity data may have drawbacks, such as recall bias.

Implications for Policy:

Based on these data, think about making specific recommendations for healthcare practitioners. How, for example, could recommendations for physical exercise be modified for senior citizens with high WWI and multimorbidity?

The forest plot visualization in Figure 2 effectively highlights the interaction effects. Ensure that the figure legends provide complete information for interpretation without referring back to the main text.

Reviewer 2

1. justification would be needed for the selection of this year and these respondents from 2011 to 2018 from the Chinese Longitudinal Healthy Longevity Survey (CLHLS)

2. The author points out that there are limitations in comparing the results with data from other countries (the CLHLS database limits generalizability to other countries.), so it would not be correct to refer to these data in the discussion.

3. Conclusion – more precise information would be needed in the conclusions.

4. It is necessary to describe the purpose of the research more precisely and provide a meaningful and reasoned answer in the conclusions, based on specific results
